# Intraabdominal Compartment Syndrome Complicating Transurethral Resection of Bladder Tumor

**DOI:** 10.1155/2012/870619

**Published:** 2012-08-21

**Authors:** Sachin Narain, Jadelis Giquel, Armando Ariza, Ricardo Martinez-Ruiz, Bruce R. Kava, Christina Matadial

**Affiliations:** ^1^Department of Anesthesiology, Perioperative Medicine and Pain Management, University of Miami Miller School of Medicine, 1611 NW 12th Avenue, Miami, FL 33136, USA; ^2^Department of Anesthesiology, Perioperative Medicine and Pain Management and Bruce W. Carter Veterans Affairs Medical Center, University of Miami Miller School of Medicine, 1611 NW 12th Avenue, Miami, FL 33136, USA; ^3^Department of Anesthesiology, Perioperative Medicine and Pain Management and Bruce W. Carter Veterans Affairs Medical Center, University of Miami Miller School of Medicine, Miami, FL 33136, USA; ^4^Division of Cardiothoracic Anesthesia, Department of Anesthesiology, Perioperative Medicine and Pain Management, Jackson Memorial Hospital and Bruce W. Carter Veterans Affairs Medical Center, University of Miami Miller School of Medicine, Miami, FL 33136, USA; ^5^Department of Urology and Bruce W. Carter Veterans Affairs Medical Center, University of Miami Miller School of Medicine, Miami, FL 33136, USA; ^6^Department of Anesthesiology, Perioperative Medicine and Pain Management and Bruce W. Carter Veterans Affairs Medical Center, University of Miami Miller School of Medicine, Miami, FL 33136, USA

## Abstract

Abdominal compartment syndrome can result from many different causes. We present a case where this dangerous syndrome occurred in the operating room during a transurethral resection of a bladder tumor. It was initially recognized by an elevation in the peak inspiratory pressure. We report the typical physiologic changes that occur with this syndrome and its treatment options.

## 1. Introduction

There is an incidence of abdominal compartment syndrome (ACS) of 5–14% of patients with severe abdominal or pelvic trauma undergoing emergency laparotomy [1, 2]. ACS most often results from blunt abdominal trauma with intra-abdominal bleeding but may even be due to the compressive effect of intra-abdominal packing [3]. Other etiologies include ruptured abdominal aortic aneurysm, liver transplantation, postpartum hemorrhagic pancreatitis, bowel obstruction, abdominal growths, ascites, and secondary ACS from massive volume resuscitation [2, 4]. ACS may lead to multi-organ failure (MOF) in as many as 36% of cases.

The initial insult produces hypovolemic shock with the subsequent increase in sympathetic outflow leading to a decrease in splanchnic perfusion. Tissue hypoxia increases the levels of proinflammatory, vasodilatory cytokines which also increase capillary permeability, causing tissue edema. Inadequate oxygen delivery also limits ATP production and impairs the sodium-potassium pump. Sodium then leaks back into cells and pulls water along leading to cellular swelling and eventually rupture and spillage of intracellular contents that then promotes further inflammation, capillary leakage, and worsening edema. After cellular reperfusion, oxygen-free radicals are generated which disrupt cell membranes. As the pressure mounts, intestinal perfusion is further impaired, and the cycle of cellular hypoxia, cell death, inflammation, and edema continues [5, 6]. The initial insult provides the “first hit,” and the subsequent cytokine release may serve as the “second hit” in progression to multiorgan failure [6].

## 2. Case Presentation

An 83-year-old male with a history of hypertension, three-vessel coronary artery bypass, and chronic obstructive pulmonary disease underwent a cystoscopy for hematuria. Rapid sequence induction and intubation were uneventful and he was placed on volume control ventilation. Shortly after the urologists began the procedure, his peak inspiratory pressure (PIP) rose, oxygen saturation dropped, and he became hypotensive and difficult to ventilate. He was placed on 100% oxygen and given albuterol and fluids along with one gram of calcium and 40 mcg of epinephrine in an initial resuscitation attempt. The urologists stopped working due to his continuing instability. His PIP rose further to 50–55 cm H_2_O with plateau pressures above 20 cm H_2_O with a tidal volume of only 300 mL on pressure control ventilation.

He was found to have a grossly rigid and distended abdomen. A cystogram showed extravesicular contrast, and an abdominal ultrasound revealed a fluid collection separating the liver from the abdominal wall and the right kidney. A drain was placed into the right upper quadrant with 1500 mL of clear fluid draining out immediately. His PIP and oxygenation improved, abdomen became less tense, and he was then transferred to the intensive care unit.

Within an hour he developed refractory hypotension, a firm, distended abdomen and became anuric. His PIP again rose above 50 cm H_2_O, and he developed ST segment changes on EKG monitoring. When there was no benefit with neuromuscular relaxation, he was taken back to the operating room for an emergent exploratory laparotomy.

At this point the PIP was above 60 cm H_2_O, and he remained markedly hypotensive despite vasopressor support. A 1.5 L intra-abdominal hematoma was evacuated along with more than 4 L of sanguinous fluid. His blood pressure immediately rose, and pressors were titrated off. A 0.5 cm bladder tear, sigmoid mesenteric tear, and arterial damage were all repaired, and a suprapubic drain was placed. The patient was switched back to volume control ventilation and now had a PIP of 28 cm H_2_O. He recovered well in the intensive care unit and was extubated 4 days later.

## 3. Discussion

This patient avoided a potential bad outcome from a very dangerous syndrome. From the rapid loss of blood into the abdominal cavity he developed an abdominal compartment syndrome that initially manifested as an elevated peak inspiratory pressure with a normal plateau pressure—suggestive of an increase in airway resistance.

The normal intra-abdominal pressure (IAP) is 5–7 mmHg in critically ill patients [7]. The development of abdominal compartment syndrome (ACS) from intra-abdominal hypertension (IAH) does not occur at a precise level. ACS is thought to develop once the abdominal pressures reach 20–25 mmHg and is characterized by an increase in airway pressures, inadequate ventilation and oxygenation, altered renal function, and hemodynamic instability [1, 2].

Each patient may have a varied presentation as to the IAP at which symptoms develop, and a rough grading system, developed by Burch, may correlate with the need for treatment. Those with Grade I disease (10–15 cm H_2_O) may begin having hemodynamic decompensation but often do not require abdominal decompression. Patients with a Grade II elevated IAP (15–25 cm H_2_O) should be closely monitored for disease progression. Grade III (25–35 cm H_2_O) and IV (>35 cm H_2_O) typically require decompression [8].

In experiments done on dogs, Barnes found that 20% of the elevated abdominal pressure was transmitted to the thoracic cavity—presumably by bulging of the diaphragm [9]. Venous return began to decrease when the IAP rose above 10 mmHg [9]. Stroke volume dropped by as much as 36% with abdominal pressures of 40 mmHg stemming from poor venous return and elevated systemic vascular resistance [6]. There was a corresponding drop in cardiac output as heart rate did not noticeably change [9]. CVP and left and right atrial pressures were artificially elevated despite the low intravascular volume. Intrathoracic pressure increases were greater than the increases in atrial or ventricular pressures; therefore, overall transmural pressures across the atrial and ventricular walls dropped.

The increase in thoracic pressure results in a decrease in all lung volumes except residual volume [9]. However the bulk of the drop comes from the expiratory reserve volume as the increased abdominal pressure overcomes the natural expansive tendencies of the rib cage [9]. As the intra-abdominal pressure rises, there is a corresponding drop in abdominal compliance, and a patient on mechanical ventilation with pressure control ventilation will begin to have smaller tidal volumes [6, 9]. If a patient is on volume control ventilation, as in this case, a precipitous rise in peak airway pressures may be seen [6].

Renal impairment is a local phenomenon caused presumably by direct renal compression and is not related to cardiac output. At an IAP of 20 mmHg renal blood flow (RBF) and glomerular filtration rate (GFR) were less than 25% of normal [10]. At an IAP of 40 mmHg RBF and GFR were 7% of normal while cardiac output was 37% of normal [10]. Clinically the urine output steadily drops toward oliguria. And activation of the renin-angiotensin-aldosterone system may lead to acute tubular necrosis and acute renal failure [6].

Once the abdominal pressure begins to rise, splanchnic blood flow begins to suffer. Blood flow through celiac and superior mesenteric arteries is reduced by 42% and 61%, respectively, with an IAP of 40 mmHg [9, 11].

Elevated intrathoracic pressures transmitted from the abdomen may impair cranial venous drainage leading to an elevated intracranial possible with potential decrease in cerebral perfusion pressure in patients with a concurrent head injury [11].

It is important to note that ACS may develop in any critically ill patient even though it most commonly follows abdominal trauma and damage control laparotomy [3]. Using just an abdominal physical exam alone is not an adequate method of detecting an elevated IAP. Kirkpatrick found that blinded examiners had both a poor sensitivity and poor accuracy in detecting an elevated IAP [12]. Urinary bladder pressure may serve as a measurement of the abdominal pressure as described by Kron et al. [13]. A pressure transducer can be connected to the Foley catheter sampling port. The pressure is measured with the patient supine and the symphysis pubis as the zero point. Other methods described include inferior vena caval pressures, nasogastric pressure transducers, and direct measurement of abdominal pressure via intra-abdominal drains.

Treatment of ACS begins with supportive measures including mechanical ventilation and careful fluid resuscitation with early goal-directed therapy. Note that excessive fluid infusion is itself a predictor of IAH [7]. Neuromuscular blockade can be used to improve the compliance of the abdominal wall and may decrease the IAP in some patients [14]. Definitive therapy is decompression, either percutaneously with a catheter or surgically.

In the presented case, the patient suffered from severe abdominal compartment syndrome based on his clinical presentation. His primary presentation was of increased peak airway pressure but soon after included most of the other stigmas of ACS: hemodynamic compromise, difficulty in ventilation and progression toward anuria. His presentation did not readily improve with supportive measures or with neuromuscular blockade, and he ultimately required decompressive surgical laparotomy as definitive treatment. In severe cases of abdominal compartment syndrome we recommend using a low threshold for surgical decompression.
